# Draft Genome Sequence of *Frankia* sp. Strain DC12, an Atypical, Noninfective, Ineffective Isolate from *Datisca cannabina*

**DOI:** 10.1128/genomeA.00889-15

**Published:** 2015-08-06

**Authors:** Louis S. Tisa, Nicholas Beauchemin, Michael N. Cantor, Teal Furnholm, Faten Ghodhbane-Gtari, Lynne Goodwin, Alex Copeland, Maher Gtari, Marcel Huntemann, Natalia Ivanova, Nikos Kyrpides, Victor Markowitz, Kostas Mavrommatis, Natalia Mikhailova, Imen Nouioui, Rediet Oshone, Galina Ovchinnikova, Ioanna Pagani, Krishnaveni Palaniappan, Amrita Pati, Arnab Sen, Nicole Shapiro, Ernest Szeto, Luis Wall, Jessie Wishart, Tanja Woyke

**Affiliations:** a University of New Hampshire, Durham, New Hampshire, USA; b Los Alamos National Laboratory, Los Alamos, New Mexico, USA; c University of Tunis-El Manar, El Manar, Tunisia; d DOE Joint Genome Institute, Walnut Creek, California, USA; e University of North Bengal, Siliguri, India; f University of Quilmes, Buenos Aires, Argentina

## Abstract

*Frankia* sp. strain DC12, isolated from root nodules of *Datisca cannabina*, is a member of the fourth lineage of *Frankia*, which is unable to reinfect actinorhizal plants. Here, we report its 6.88-Mbp high-quality draft genome sequence, with a G+C content of 71.92% and 5,858 candidate protein-coding genes.

## GENOME ANNOUNCEMENT

*Frankia* spp. are well known as plant symbionts of dicotyledonous plants and also are found as free-living soil dwellers (1–3). This genus has not yet been described to the species level, but it has become an area of recent interest. Four major *Frankia* lineages have been identified (4–7). Three of them are known to reinfect their host plant, while the fourth lineage (termed atypical *Frankia* isolates) are unable to reinfect actinorhizal plants or will reinfect the host plant but form ineffective nodules. Our understanding of this genus has been greatly enhanced by the sequencing of several *Frankia* genomes from the different *Frankia* lineages (8–18).

As a member of the fourth *Frankia* lineage, *Frankia* sp. strain DC12 was chosen for sequencing. This atypical noninfective (Nod^−^) and non-nitrogen-fixing (Fix^−^) *Frankia* strain was isolated from root nodules of *Datisca cannabina* L. collected from Swat, Pakistan (19, 20). Strain DC12 is also resistant to elevated levels of toxic heavy metals (21), and the spores germinate well under controlled conditions, enabling single genomic units to be isolated (22). Strain DC12 was sequenced to provide information about the potential ecological roles of the atypical *Frankia* strains and interaction with actinorhizal plants.

The draft genome of *Frankia* sp. strain DC12 was generated at the Department of Energy (DOE) Joint Genome Institute (JGI) using Illumina data (23). An Illumina short-insert paired-end library with an average (± standard deviation) insert size of 242 ± 59 bp, which generated 16,229,834 reads, and an Illumina long-insert paired-end library with an average insert size of 6,525 ± 1,400 bp, which generated 20,981,340 reads totaling 4,533 Mbp of Illumina data, were generated and sequenced. All techniques for DNA isolation, library construction, and sequencing were performed according to JGI standards and protocols (http://www.jgi.doe.gov). The Illumina sequencing data were assembled with Velvet version 1.0.13 (24) and AllPaths version r41043 (25). The final draft assembly contained 12 contigs in 1 scaffold. The total size of the genome is 6.88 Mbp, and the final assembly is based on 4,533 Mbp of Illumina draft data, which provides an average 657× coverage of the genome. For finishing, the gaps and misassemblies were resolved by editing in Consed, PCR, and sequencing of bridging PCR fragments with Sanger and/or PacBio technologies.

Project information is available in the Genomes Online Database (26). Genes were identified using Prodigal (27), followed by a round of manual curation using GenePRIMP (28) as part of the microbial annotation pipeline of the JGI (29). Additional gene prediction analysis and manual functional annotation were performed within the Integrated Microbial Genomes-Expert Review (IMG-ER) platform (http://img.jgi.doe.gov) developed by the Joint Genome Institute (Walnut Creek, CA, USA) (30).

The high-quality draft genome of *Frankia* sp. DC12 was resolved to 1 scaffold consisting of 6,884,336 bp, with a G+C content of 71.92%, 5,858 candidate protein-coding genes, 46 tRNA genes, and 3 rRNA regions.

### Nucleotide sequence accession numbers.

The *Frankia* sp. strain DC12 genome sequence has been deposited at DDBJ/EMBL/GenBank under the accession no. LANG00000000. The version described in this paper is the first version, LANG01000000.
